# From IgZ to IgT: A Call for a Common Nomenclature for Immunoglobulin Heavy Chain Genes of Ray-Finned Fish

**DOI:** 10.1089/zeb.2021.0071

**Published:** 2021-12-15

**Authors:** Alex Dornburg, Tatsuya Ota, Michael F. Criscitiello, Irene Salinas, J. Oriol Sunyer, Susana Magadán, Pierre Boudinot, Zhen Xu, Martin F. Flajnik, Amy Singer, Francisco Gambón-Deza, John D. Hansen, Jeffrey A. Yoder

**Affiliations:** ^ 1^ Department of Bioinformatics and Genomics, University of North Carolina at Charlotte, Charlotte, North Carolina, USA.; ^2^ Department of Evolutionary Studies of Biosystems, SOKENDAI (The Graduate University for Advanced Studies), Hayama, Japan.; ^3^ Comparative Immunogenetics Laboratory, Department of Veterinary Pathobiology, College of Veterinary Medicine and Biomedical Sciences, Texas A&M University, College Station, Texas, USA.; ^4^ Department of Biology, Center for Evolutionary and Theoretical Immunology (CETI), University of New Mexico, Albuquerque, New Mexico, USA.; ^5^ Department of Pathobiology, School of Veterinary Medicine, University of Pennsylvania, Philadelphia, Pennsylvania, USA.; ^6^ Centro de Investigaciones Biomédicas, Universidade de Vigo, Campus Universitario Lagoas Marcosende, Vigo, Spain.; ^7^ Université Paris-Saclay, INRAE, UVSQ, Virologie et Immunologie Moléculaires, Jouy-en-Josas, France.; ^8^ Institute of Hydrobiology, Chinese Academy of Sciences, Wuhan, China.; ^9^ Department of Microbiology and Immunology, University of Maryland Baltimore School of Medicine, Baltimore, Maryland, USA.; ^10^ Zebrafish Nomenclature Coordinator, Zebrafish Model Organism Database (ZFIN), University of Oregon, Eugene, Oregon, USA.; ^11^ Unidad de Inmunología Hospital do Meixoeiro, Vigo, Spain.; ^12^ U.S. Geological Survey, Western Fisheries Research Center, Seattle, Washington, USA.; ^13^ Department of Molecular Biomedical Sciences, North Carolina State University, Raleigh, North Carolina, USA.

Ray-finned fishes comprise more than half the ∼60,000 known vertebrate species,^1^ and are pivotal to the functionality of aquatic ecosystems and success of global multibillion dollar industries. Understanding ray-finned fish immune systems is essential to predicting how species will respond to known or emergent pathogens as well as to the development of effective vaccines for aquaculture. However, the diversity of species, including in aquaculture, necessitates that immunology and translational medicine research groups investigating the immune system in one or a number of species employ a common language for describing homologous immune components. Unfortunately for Immunoglobulin (Ig) genes that encode antibodies, this has not been the case.

**FIG. 1.**
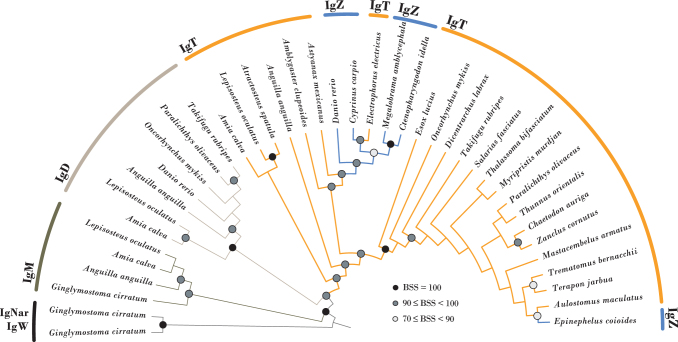
IgT and IgZ are evolutionary forms of the same antibody isotype. A maximum likelihood estimate of the phylogenetic relationships of representative IgT (*orange*) and IgZ (*blue*) sequences. The C-terminal constant domain of each Ig was aligned using MAFFT. A maximum likelihood based on phylogeny was estimated in IQ-TREE 2,^9^ and conditioned on the best-fit model of amino acid substitution selected by Bayesian information criterion. Node support was assessed through 1000 bootstrap replicates and BSS is indicated by *shaded circles* at nodes. One hundred percent BSS: *solid black*; 90%≤ BSS <100%: *dark gray*; 70% ≤ BSS <90%: *light gray*; BSS <70%: *no circle*. IgT and IgZ sequences were described previously^8^ or listed here: bowfin (*Amia calva*, scaf18:9770670-9770347), spotted gar (*Lepisosteus oculatus*, JH591552.1:10457-10113), European eel (*Anguilla anguilla*, NC_049202.1:63242796-63243128), zebrafish (*Danio rerio*, AAT67446.1), rainbow trout (*Oncorhynchus mykiss*, AAW66978.1), Japanese pufferfish (*Takifugu rubripes*, BAD69712.1), and olive flounder (*Paralichthys olivaceus*, ANS12795.1). IgM sequences (*olive green*) included: spotted gar (LG5:577726-578115), bowfin (scaf18:10347166-10347555), European eel (ABY73532.1), and nurse shark (*Ginglymostoma cirratum*, AAA50817.1). IgD sequences (tan) included: Japanese pufferfish (BAD34541.1), olive flounder (BAB41204.1), rainbow trout (AAY41237.1), zebrafish, (Chr3:33950441-33950157), European eel (NC_049202.1:63885951-63886238), spotted gar (LG5:605556-605846), and bowfin (scaf18:10369108-10368818). Nurse shark IgW (AAB08972.1) and IgNar (AAB42621.2) were employed as outgroups (*black*).^7^ BSS, bootstrap support.

Ig genes are restricted to jawed vertebrates (gnathostomes) with all lineages encoding common heavy chains IgM and IgD (aka IgW in cartilaginous fish, lungfish, and coelacanths).^2^ Before 2005, it was believed that bony fish encoded only IgM and IgD. In 2005, Hansen et al. described a new Ig heavy chain encoded within the rainbow trout heavy chain locus and named it IgT for “teleost.”^3^ However, during the same year, Steiner and colleagues described a new heavy chain within the heavy chain locus of zebrafish and named it IgZ, presumably for zebrafish.^4^ As these projects were being published, it became clear that IgT and IgZ encode not only very similar sequences (see note in^3^) but are likely “evolutionary forms of the same antibody” isotype (Fig. 1).^5^

In addition, the conserved organization of the heavy chain locus in many teleost species (with a basic scheme of D_T_J_T_C_T_ or D_Z_J_Z_C_Z_ gene segments between sets of V and D_M_J_M_C_M_/C_D_ gene segments) supports orthology between IgT and IgZ.^3,4^ This heavy chain sequence has since been identified in a large number of ray-finned fish species, and shown to play important roles in mucosal immunity,^6^ with many species adopting the IgT nomenclature and others (especially within cyprinids) using IgZ. Consequently, it has become routine for many authors to refer to this sequence in publications as IgT/Z.

To increase consistency in vertebrate immunogenetics, we propose a single nomenclature system is warranted for this heavy chain. IgZ continues to be used in zebrafish, a powerful model for human disease and the first fish with a reference genome. However, this convention is at odds with research that spans the remaining diversity of >30,000 additional species of ray-finned fishes. A simple PubMed search (https://pubmed.ncbi.nlm.nih.gov/) using the terms “IgT antibodies” and “IgZ antibodies” identified 268 publications using IgT and only 37 using IgZ (search ran on October 1, 2021), suggesting that IgT is either more prevalent across species, or more reports are published from species with a history of using IgT. This trend will surely accelerate given the rapid growth of genomic resources for non-model species, rendering IgZ a source of potential future confusion in comparative studies.

We recognize that ideally, either IgT or IgZ would reflect an inclusive name. However, recent identification of IgT/Z from holostei (the sibling lineage of teleosts) demonstrates that this class of antibody extends outside of teleosts.^7,8^ As a consequence, neither IgT nor IgZ is completely inclusive if we rely on the T as referring to “teleost-specific” and Z referring to “zebrafish-specific.” Regardless of this discrepancy in nomenclature, we feel that IgT remains the most appropriate choice as IgZ reflects a history of more taxonomically restricted usage and IgT has already been adopted in non-teleost species.

Therefore, discussions with the Zebrafish Nomenclature Committee have led to the changing of the official zebrafish gene symbol for the IgZ heavy chain (ZDB-GENE-040513-8) from immunoglobulin heavy constant zeta (*ighz*) to immunoglobulin heavy constant tau (*ight*). We now encourage a shift from IgZ to IgT in all ray-finned fish species.
